# Evaluation of Leptin and Adiponectin Levels in Patients with Stable Angina Pectoris

**Published:** 2010

**Authors:** Masoud Mozafari, Masoumeh Sadeghi, Hamid Sanei, Mohammad Arash Ramezani, Allahyar Golabchi, Nizal Sarrafzadegan

**Affiliations:** 1 Cardiology Resident, Isfahan University of Medical Sciences, Isfahan, Iran; 2 Associate Professor of Cardiology, Isfahan Cardiovascular Research Center, Isfahan University of Medical Sciences, Isfahan, Iran; 3 Associate Professor of Cardiology, Isfahan University of Medical Sciences, Isfahan, Iran; 4 Community Medicine Specialist, Isfahan Cardiovascular Research Center, Isfahan, Iran; 5 Professor of Cardiology, Isfahan Cardiovascular Research Center, Isfahan University of Medical Sciences, Isfahan, Iran

**Keywords:** Leptin, Adiponectin, Coronary artery disease

## Abstract

**BACKGROUND:**

Leptin and adiponectin are two adipose tissue hormones and their association with the incidence of cardiovascular diseases is under evaluation. The aim of this study was to determine the relationship of leptin and adiponectin with coronary artery diseases.

**METHODS:**

One hundred and seventy patients with angina pectoris and indications of coronary angiography underwent angiography. Serum levels of blood lipids, leptin, and adiponectin were measured. The gathered data was evaluated using SPSS_15_ software, by multivariate variance analysis.

**RESULTS:**

Analysis of the data demonstrated that 45.1% of the patients had positive angiographic findings. The serum levels of leptin and adiponectin were significantly lower than the minimum levels specified by the kit. However, the two groups, i.e., patients with positive angiographic findings and those with negative findings were not significantly different according to the serum levels of the hormones. Moreover, no significant correlation between the serum levels of the hormones and serum lipids was observed.

**CONCLUSION:**

Various studies have demonstrated that high serum level of leptin and the incidence of coronary artery diseases are correlated. On the other hand, they have reported that adiponectin has cardioprotective role. Confirmation of these findings requires more detailed studies.

## Introduction

Today, cardiovascular diseases (CVD) are among the major causes of mortality throughout the world, and a significant load of the diseases belongs to CVD.^1^, ^2^ Many preventive programs have focused on the control of these diseases, and the most important ones are addressing the issue of identification and reduction of the risk factors of CVD incidence. Main known risk factors for CVD are obesity, hypertension, diabetes, and hyperlipidemia.^3^ Recent studies are seeking new and critical risk factors in incidence of ischemic heart diseases. Some of these factors are lipoprotein Apo A and Apo B. However, some chemical mediators have been discovered in blood, which are correlated with atherosclerosis and the incidence of coronary artery diseases (CAD). Leptin and adiponectin are two of these chemical molecules, secreted by adipose tissue. Leptin is an amino acid discovered in 1994. Leptin deficiency is reported to be accompanied by insulin resistance, obesity, diabetes, and even infertility in experimental models and human.^4^–^6^ Leptin receptors exist in many tissues and play an important role in metabolism of lipids and carbohydrates, as well as reproduction system, and immune and inflammatory reactions.^7^ The role of leptin in the atherosclerosis phenomenon is probably one of its significant roles. The probable roles of leptin are as follows: First, many obese individuals have a hypothalamic resistance to leptin, which leads to an increase in leptin serum level, hyperleptinemia. By causing endothelial dysfunction, oxidative stress, platelet aggregation, and inflammatory reactions, hyperleptinemia will lead to atherosclerosis.^8^ Second, secretion of leptin by adipocytes is accompanied by an increase in insulin level, and insulin resistance. The relationship between hyperinsulinemia and insulin resistance, and atherosclerosis has been confirmed.^9^, ^10^ Finally, it seems that leptin causes increased sympathetic nervous system activity. Consequently, this leads to high blood pressure and diabetes, which in turn causes an increase in incidence of atherosclerosis and CVD.^11^, ^12^ Adiponectin is opposite of leptin in many aspects. Adiponectin is also a hormone secreted from adipose tissue, which in contrast to leptin plays an anti-atherogenic role. However, its serum level is lower in obese individuals, compared with those with normal body weight. It leads to increased sensitivity to insulin^13^, ^14^ and its blood level is higher in patients with Type 2 Diabetes and essential hypertension.^15^–^17^ Adiponectin receptors are found in peripheral tissues of skeletal system, GI system, liver, and endothelial cells.^18^ By inhibiting the effect of plaque forming inflammatory factors in endothelial cells, adiponectin prevents development and progression of atherosclerosis, and considered a protective factor against CAD.^15^ The relationship between secretion and physiological function of leptin and adiponectin has not been defined, but the important issue is the ratio of leptin to adiponectin, which is considered as a risk factor for CAD.^19^

Nevertheless, most data were obtained in laboratory settings and there are few clinical data available in this regard. The objective of this study was to determine the relationship between the serum level of leptin and adiponectin in patients with ischemic heart diseases, as well as evaluation of the relationship between serum lipids and the two hormones.

## Method and Materials

This was a cross-sectional study carried out on 170 patients with stable angina pectoris, referred to Isfahan hospitals (Korshid Hospital and Shahid Chamran Hospital). Before beginning of the study, sample size was determined according to appropriate statistical formulations. The patients were selected by easy sampling method from the angiography ward of the above-mentioned hospitals. Before performing coronary angiography study, each patient attended an interview session and his/her demographic data including age, sex, occupation, education level, living place, marital status, and the work and home addresses were recorded. Then, the patients underwent physical examination and their height, weight, and blood pressure were measured. The patients’ height was measured with bare foot using a height bar and the weight was measured using a calibrated standard scales. Patients’ blood pressure was measured by a calibrated sphygmomanometer with adult cuff in lying position from their right hand. The patients were asked to avoid having coffee and tea before physical examination, taking rest for at least 30 minutes, and not being anxious. Blood pressure was measured according to standard blood pressure measurement guideline.^18^ The 10 cc venous blood sample taken from cubital vein of each patient was sent to the laboratory of Isfahan Heart Research Center for laboratory evaluations. In the laboratory, blood cholesterol and triglyceride (TG) were measured by Autoanalyzer (Hitachi), using enzymatic method, high density lipoprotein (HDL) by precipitation with dextran sulfate, and low density lipoprotein (LDL) by Freidwald formulation method for patients with TG<400 and by direct method for those with TG>400. Also, leptin and adiponectin were automatically measured by enzymatic method using proper kits (Pars Azma Co., Iran). Laboratory findings of each patient were recorded in a particular form. Then, the patients were referred for coronary angiography and underwent complete coronary artery angiography. Involvement of any of the arteries including left main (LM), left anterior descending (LAD), left circumflex (LCx), right coronary (RC), and posterior descending (PD) arteries, and all their branches as well as the percentage of their involvement were recorded. Involvement percentage higher than 50% in LM was scored two and involvement of LAD, LCx, RCA, PDA, and their branches higher than 75% was scored one. If a patient had LM involvement, involvement of other arteries was not regarded. The overall arterial involvement was measured by multiplying the scores. The data was analyzed by SPSS_15_ software. The numerical data and nominal data were described using mean and standard deviation, and frequency percentage, respectively, and t-test was used for mean comparisons. Considering the leptin and adiponectin alterations in different ages in both genders, linear variance analysis and ANCOVA models were used to eliminate the effect of age and gender. Pearson's correlation coefficient was used to evaluate the correlation among numerical variables. Level of significance was considered to be less than 0.05 in all tests.

## Results

Of 170 participants, six patients were excluded due to incomplete demographic and laboratory data; 74 patients (45.1%) of 164 cases were male. Mean age of the cases was 53.4 ± 7.6 years. The demographical characteristics of patients are presented in Table 1.

**Table 1:** Demographics characters of patients.

Variables	Frequency	Percent
Sex		
Female	74	45.1%
Male	90	54.9%
Age mean (SD)	53.7 (7.6)	
Marital Status	1
Single	137	0.6%
Married	5	83.5%
Divorced	21	3%
Widow		12.8%
Job		
Employer	13	8%
Worker	18	11%
Self-employed	30	18.3%
Housekeeper	86	52.4%
Unemployed and Retired	17	10.3%
Education	51	
Illiterate	87	31.1%
Elementary	20	53%
Diploma	6	12.2%
University educated		3.7%

Only 74 patients (45.1%), of all the cases who had stable angina and were candidate of coronary angiography had signs of coronary artery stenosis and 90 patients did not have any signs indicative of coronary artery stenosis. Mean serum level of lipids and apo-lipoproteins A and B of the two groups are separately provided in Table 2. It was demonstrated that the mean level of blood lipids in the group with negative angiographic findings were lower compared with that of the group with positive findings. Comparison of the serum leptin and adiponectin levels as well as the ratio of leptin to adiponectin did not show any significant difference between the two groups. The two groups were significantly different only regarding serum levels of total cholesterol, TG, and apo-lipoproteins A and B. It should be noted that the mean level of both leptin and adiponectin in patients was significantly lower than the minimum level specified for the kit; i.e., 15.1 ng/ml versus 26 ng/ml as the minimum level for leptin and 10.1 µg/ml versus 14 µg/ml for adiponectin. Pearson's correlation coefficient was employed to evaluate the relationship among leptin and adiponectin, and blood levels of lipids. The test did not find any correlation in this regard. The correlations between the above-mentioned variables are provided in Table 3. Considering the alteration of leptin and adiponectin level in different ages in two genders, linear variance analysis model ANCOVA was used to eliminate the effect of age and gender variables. However, the ratio of serum level of leptin to adiponectin and serum level of leptin and adiponectin was not significantly different between the two groups with positive and negative angiographic findings. Table 4 demonstrates the age and gender modified ratio of leptin to adiponectin.

**Table 2:** Distribution of blood lipids in the two groups of study.

variable	group	Mean (SD)	t-test	P
TC (mg/dl)	Angiography (−)	200 (43.77)	2.88	0.005
	Angiography (+)	179.28 (47)		
TG(mg/dl)	Angiography (−)	194.67 (122.46)	2.042	0.04
	Angiography (+)	159.79 (93.35)		
	Angiography (−)	115 (31.46)		
LDL(mg/dl)	Angiography (+)	106.36 (33)	1.697	0.09
HDL(mg/dl)	Angiography (−)	32 (12.09)	1.976	0.05
	Angiography (+)	35.56 (10.34)		
Apo-A	Angiography (−)	157.17 (26.94)	2.245	0.03
	Angiography (+)	147.69 (26.16)		
Apo-B	Angiography (−)	106.55 (25.63)	2.025	0.04
	Angiography (+)	97.73 (29.4)		

**Table 3:** Correlation coefficients between blood parameters and leptin and adiponectin.

Variable	Leptin	Adiponectin	Leptin/Adiponectin
Chol (mg/dl)	0.08	0.17*	−0.009
TG (mg/dl)	0.12	0.001	0.06
LDL (mg/dl)	−0.02	0.12	−0.05
HDL (mg/dl)	−0.04	0.31*	−0.12
Apo A (mg/dl)	0.16*	0.15	0.07
Apo B (mg/dl)	0.05	0.08	0.04

* P < 0.05

**Table 4:** Comparison of serum leptin and adiponectin in both groups with positive and negative angiography.

	Groups	t-test	Age and Sex Adjusted
Leptin (ng/ml)	Angiography (+)	15±1.43	14.52±1.4
	Angiography (−)	15.34±1.8	14.68 1.53
Adiponectin (µg/ml)	Angiography (+)	10.89±1	11.21±0.94
	Angiography (−)	9.23±0.91	8.88±1.02
Leptin/ Adiponectin	Angiography(+)	2.22±0.34	2.19±0.34
	Angiography (−)	2.66±0.4	2.59±0.37

*No significant difference

## Discussion

The results obtained in this study demonstrated that the mean serum level of leptin and adiponectin in the patients under study were significantly lower than the normal mean values specified in the standard kit. Nonetheless, the two groups, groups with positive and negative angiographic findings, were not significantly different, even after being age and gender modified. Physiological studies in animal models and human demonstrated the independent effect of leptin and adiponectin hormones on atherosclerosis.^19^ These studies considered a greater effect for leptin compared with adiponectin or at least have considered the ratio of leptin to adiponectin as an independent risk factor for vascular dysfunction.^12^

In a clinical study, Dubey et al. confirmed that the serum level of leptin is patients with heart attack was significantly higher than that in patients with stable angina and normal people. They also demonstrated that leptin has significant correlation with inflammation mediators contributing to atherosclerosis. Serum level of leptin has significant positive correlation with C-reactive protein and interleukin 6.^20^ Previous studies have shown that inflammatory mediators have an important and confirmed role in formation of atherosclerotic plaques. An increase in these mediators will lead to an increase in the incidence of CVD and these inflammatory mediators are considered as independent risk factors for CAD.^21^ Being familiar with the effect of leptin on CAD, Wolk et al. carried out a study on the prognosis of patients with myocardial infarction (MI) in the US. They concluded that leptin level has a direct effect on the prognosis pf MI patients and those with higher levels of leptin had coronary artery involvement higher than 50% and also their number of involved arteries was higher.^22^ In contrast to Wolk's results, our results did not show a correlation between leptin serum level and the level of coronary artery involvement. The difference possibly originates from the difference between the types of studies. Our study was cross-sectional and not longitudinal. Furthermore, we did not follow the patients’ prognoses and our only outcome was the data obtained by coronary angiography. Since the levels of these two mediators were significantly lower than those in normal population, as specified by the kit, the following points should be considered. First, there is an important relationship among inflammatory mediators and the two hormones. A limitation of current studies, including the present study is not measuring the levels of these inflammatory mediators. Common relationship among the mediators and controlling the effect of each one may lead to more accurate results.

Additionally, all participants of this study had indication for coronary angiography by obvious clinical findings. Although we categorized the patients into the two groups of positive and negative angiographic findings, the classification was merely based upon involvement of coronary arteries specified in coronary angiography. Patients with normal coronary angiography may have involvement of subepicardial or small vessels which cannot be found in coronary angiography study. It is better to carry out longitudinal studies to evaluate the effects of leptin and adiponectin. By following up the patients and performing repeated measurement of the two hormones in such studies, and also recording vascular events occurred during the patients’ follow-ups, a more clear relationship among the levels of the hormones and CAD will be found. It was demonstrated that serum leptin level increases significantly after MI, and the increase is detectable during the acute phase of MI.^23^ Recent studies have shown that adiponectin does not have a significant effect on heart diseases and there is not a relationship between adiponectin level and incidence and prognosis of coronary heart diseases.^24^–^26^ Moreover, different studies have confirmed leptin as a strong predictor in cerebral vascular accidents (CVA), and have reported that serum leptin level is higher than normal in CVA patients.^27^–^29^ Considering adiponectin, it should be noted that the higher levels of the hormone leads to the higher level of disability after CVA, which is in contrast with findings of other studies.^30^ Regarding the previous studies and the results of the present study, it is recommended to carry out longitudinal studies on CVD including patients with MI and CVA with repeated measurement of leptin and adiponectin. Results of cohort studies can clarify the relationship of the levels of the two chemical mediators and atherosclerosis.
